# Clove Essential Oil and Eugenol as Natural Antifungal Agents to Reduce Postharvest Losses in Melon (*Cucumis melo*)

**DOI:** 10.3390/ijms26199603

**Published:** 2025-10-01

**Authors:** Silvia Giménez-Santamarina, Natalia Torres-Pagan, Silvina Larran, Josefa Roselló, M. Pilar Santamarina

**Affiliations:** 1 Departamento de Ecosistemas Agroforestales, Universitat Politècnica de València (UPV), Camino de Vera s/n, 46022 Valencia, Spain; silvia.santam@gmail.com (S.G.-S.); jrosello@upvnet.upv.es (J.R.); 2 Instituto Agroforestal Mediterráneo (IAM), Universitat Politècnica de València (UPV), Camino de Vera s/n, 46022 Valencia, Spain; natorpa@etsiamn.upv.es; 3 Centro de Investigaciones de Fitopatología (CIDEFI), Facultad de Ciencias Agrarias y Forestales, Universidad Nacional de La Plata, La Plata 1900, Argentina; silvinalar@gmail.com

**Keywords:** clove essential oil, eugenol, melon “vedrantais”, melon “makuwa”, *Fusarium* spp.

## Abstract

Melon is a global crop with a value of USD 31 billion. However, up to 30% of yield is lost due to phytopathogens. Essential oils are a sustainable approach to crop protection and storage, enhancing food security and reducing agricultural losses. We evaluated the antifungal potential of clove essential oil and pure eugenol against *Alternaria alternata*, *Curvularia hawaiiensis*, *Fusarium oxysporum* f. sp. *lycopersici* (FOL), *Fusarium solani* f. sp. *cucurbitae* (FSC), *Rhizoctonia solani*, and *Verticillium dahliae* in vitro. We also evaluated the resistance of melons, including eugenol-poor *Cucumis melo* cv. *vedrantais* (CMV) and eugenol-rich *C. melo* cv. *makuwa* (CMM), to infection caused by FOL and FSC. Chemical analysis of clove oil reveals that eugenol was the main compound, at 89.28%. Clove oil and eugenol at 300 μg/mL reduced the growth of all fungal species. Pure eugenol exhibited the strongest antifungal activity, with 95–100% growth inhibition. Eugenol-rich melons did not show necrosis or internal rot when inoculated with FSC, and had minimal lesions, while eugenol-poor melons revealed more advanced rot symptoms. Clove oil and eugenol are antifungal alternatives that may improve food safety. These findings demonstrate the high potential of eugenol to reduce postharvest losses in melon and contribute to future breeding programmes aimed at developing contamination-resistant cultivars.

## 1. Introduction

The Cucurbitaceae family is one of the most diverse botanical families, with around 1000 species, including only 33 cultivated species, of which 10 are economically important [1]. Archaeobotanical evidence shows their presence in ancient cultures through the remains of leaves, seeds and fruits, with ancient texts and illustrations recording the spread of cucurbit crops from India or the Americas to the Mediterranean and northern Europe [1,2].

Cucurbits, known for their edible fruits, are grown for human consumption and are characterized by a wide range of characteristics. They can be consumed ripe or unripe, fresh or pickled, baked, cooked, and candied, and are also used in traditional medicine for their important antioxidant, anti-diabetes, anti-inflammatory, anti-cancer, and purgative properties [3,4].

The genera *Cucurbita*, *Cucumis*, and *Citrullus* are the most economically valued species within the *Cucurbitaceae* family in the global food industry and include cucumbers and melons, pumpkins and squash, and watermelons, respectively [3].

*Cucumis melo* L., commonly known as melon, sweet melon, muskmelon, or cantaloupe, among other things, is a globally cultivated fruit valued for its nutritional and bioactive properties [5]. This polymorphic species shows a large variation in fruit morphology and taste. Its versatility allows for consumption as fresh fruit, juice production, and edible seeds, while peels and seeds are also processed into extracts, flours, and oils rich in phytochemicals [6]. It is highly consumed during warm seasons for its refreshing qualities, due to its high water content (92%) and various uses in traditional medicinal, including its diuretic and purgative properties. Melons are rich in vitamin C, provitamin A, and essential minerals, with deep orange varieties offering greater antioxidant benefits [7].

Global melon production reached 28 million tons in 2021, with China as the leading producer, followed by India [8]. Melons are considered one of the primary fruit categories in Europe. Spain is a key producer across regions like Castilla-La Mancha, Murcia, Andalucía, and the Valencian Community. These regions are the top melon-producing areas, contributing up to 91% of Spain’s 524.035 ton total melon production [9], which includes different cultivations: fruits with smooth rinds (e.g., Tendral), those with reticulated green rinds (e.g., Piel de sapo), and cantaloupes (e.g., Charentais, Vedrantais, Galia), among others.

In the Valencian Community, melon cultivation covered 16.221 hectares, producing 35.108 tons in 2022 and contributing to about 7% of national production [10].

Melon production is often reduced by a great diversity of pests and diseases, the latter being caused by viruses, bacteria, fungi, and other pathogens. They affect different plant organs, causing leaf spots, wilting, rot, and fruit deformation during crop cultivation and postharvest along the food chain, causing significant yield and quality losses. Fungal diseases are considered one of the most important threats to melon crops. Many species belonging to different genera have been reported to cause plant disease and fruit rot in melon, with the most important including *Alternaria*, *Botrytis*, *Cladosporium*, *Fusarium*, *Verticillium*, *Didymella*, *Phytophthora*, *Corynespora*, *Colletotrichum*, *Pseudoperonospora*, *Erysiphe*, *Sphaerotheca*, *Macrophomina*, *Rhizoctonia*, and *Sclerotinia*, among others [11,12,13,14,15]. Moreover, species belonging to the genera *Penicillium*, *Cladosporium*, *Geotrichum*, *Rhizopus*, and *Mucor* cause decay and surface lesions that affect melon’s sensory qualities [16].

Fungal contamination of fruits and vegetables throughout the food chain, mainly by *Fusarium*, *Bipolaris*, *Curvularia*, and *Alternaria* species, causes significant postharvest losses and poses health risks due to mycotoxin production and opportunistic pathogenicity [17,18,19]. Species of the genera *Fusarium* have been reported in diverse melon-producing regions globally and are acknowledged as significant postharvest pathologies affecting fruit quality and marketability. Recently, species belonging to the *Fusarium incarnatum–equiseti* species complex (FIESC), the *Fusarium solani* species complex (FSSC), the *Fusarium oxysporum* species complex (FOSC), and the *F. fujikuroi* species complex (FFSC) have been reported to cause fruit rot in melon in Thailand, China, and Brazil [12].

Chemical fungicides are frequently used to reduce pathogens but pose health and environmental risks, and can lead to resistant strains. Therefore, there is growing interest in biological control and safer alternatives, such as natural compounds like essential oils, which show promise as antioxidants and antifungals for managing crop and postharvest diseases. Essential oils are proposed as effective natural alternatives to synthetic fungicides due to their antifungal properties [20,21,22,23,24,25].

Clove essential oil (EO) [*Syzygium aromaticum* (L.) Merr. & L.M. Perry] and its main component, eugenol, have shown antioxidant, analgesic, and anti-cancer properties, and recently have been used as natural food preservatives [26]. Research on clove oil and eugenol has demonstrated their potential as functional compounds in films, enabling the development of packaging with antioxidant and antimicrobial properties for food preservation [27,28,29]. They also exhibited antifungal effects against *Aspergillus flavus*, a seedborne spoilage fungus on wheat grain at the postharvest stage, as reported by Quin et al. [30]. Additionally, previous studies conducted by our research team have demonstrated the antifungal activity of clove EO activity against several phytopathogenic fungi on Mediterranean rice grains and postharvest wheat, including *A*. *alternata*, *Bipolaris oryzae*, *Fusarium graminearum*, *Fusarium equiseti*, and *Fusarium verticillioides* [31].

Despite these promising findings, no study has yet evaluated the efficacy of clove EO or eugenol against phytopathogenic fungi affecting melons during postharvest storage. This highlights the relevance of the present research, which seeks to explore these natural compounds as sustainable alternatives for managing postharvest crop diseases.

Interestingly, high amounts of the phenolic compound eugenol have been identified in different types of melons, such as in the skin of pocket melon (*C. melo* ssp. *dudaim*), in the peels and seeds of *C. melo* var. *reticulatus*, and in the flesh of Piel de sapo (*C. melo* cv. Trujillo) and *Makuwa*, which contribute to their aromatic profiles [32,33,34,35]. However, to date, there is a lack of studies evaluating the role of endogenous eugenol in melon resistance to postharvest pathogens. Given the well-documented antifungal properties of eugenol, this study aims to investigate whether melons with high endogenous levels of eugenol exhibit increased resistance to postharvest fungal pathogens.

In the current study, our objectives were to (i) determine the chemical composition of clove (*Syzygium aromaticum*) essential oil; (ii) evaluate the antifungal potential of clove essential oil and pure eugenol against six pathogenic fungi in an in vitro assay; and (iii) assess the resistance of *Cucumis melo* subsp. *melo* var. *cantalupensis* cv. *vedrantais* (CCV) and *C. melo* subsp. *agrestis* var. *makuwa* cv. *makuwa* (CCM) melons to infection caused by *Fusarium oxysporum* f. sp. *lycopersici* and *Fusarium solani* f. sp. *cucurbitae*.

## 2. Results

### 2.1. Chemical Composition of Clove Essential Oil (EO)

The chemical components of commercial clove obtained based on Gas Chromatography–Mass Spectrometry (GC/MS) analysis are shown in Table 1. Each compound was identified using mass spectrometry (MS), and its identity was confirmed by comparing its retention indices (RIs) and mass spectra with those of authentic standards or with reference data from the National Institute of Standards and Technology (NIST) 2005 Mass Spectral Library.

Eighteen compounds, representing 99.54% of the total clove essential oil, were identified. The main components were aromatic compounds (89.48%), sesquiterpene hydrocarbons (8.06%), and oxygenated sesquiterpenes (0.92%). The phenylpropanoid eugenol was the main aromatic compound found, accounting for 89.28%, while others were detected in insignificant amounts (0.54%) (Table 1).

### 2.2. Antifungal Activity of the Syzygium aromaticum EO and Eugenol

#### 2.2.1. Effect of Clove EO and Eugenol on Fungal Growth Rates

**Figure 1:**
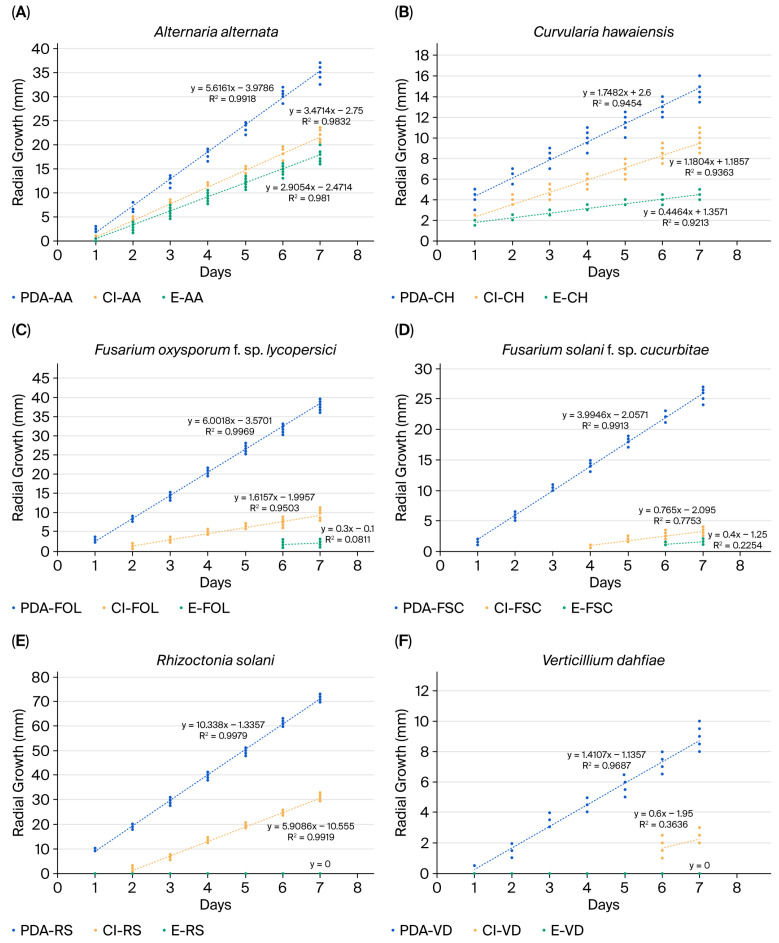
Growth rates (mm·day^−1^) of (**A**) *Alternaria alternata*, (**B**) *Curvularia hawaiiensis*, (**C**) *Fusarium oxysporum* f. sp. *lycopersici*, (**D**) *Fusarium solani* f. sp. *cucurbitae*, (**E**) *Rhizoctonia solani*, and (**F**) *Verticillium dahliae* on clove essential oil (Cl), pure eugenol (E), and potato dextrose agar (PDA).

**Figure 2:**
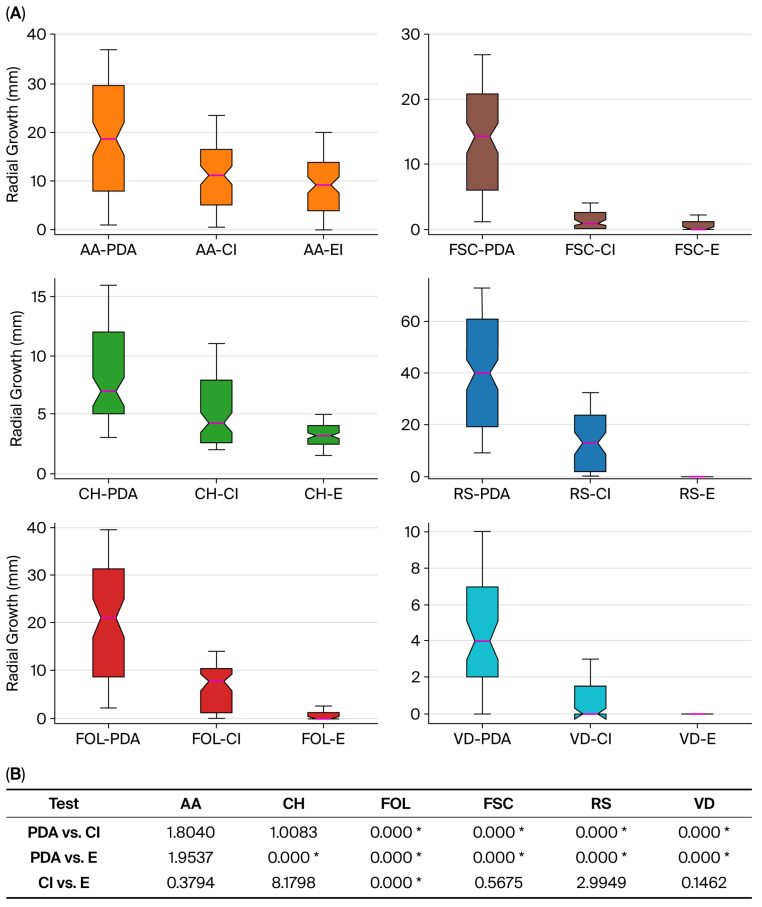
(**A**) Boxplots representing the data distribution, mean (purple line), and quartiles for control (PDA) and clove (Cl)- and pure eugenol (E)-treated samples, both at 300 µg/mL, against *Alternaria alternata* (AA, orange), *Curvularia hawaiiensis* (CH, green), *Fusarium oxysporum* f. sp. *lycopersici* (FOL, red), *Fusarium solani* f. sp. *cucurbitae* (FOS, brown), *Rhizoctonia solani* (RS, blue), and *Verticillium dahliae* (VD, light blue); (**B**) ANOVA analysis results. *p*-values obtained from Tukey’s HSD test at a 95% confidence level. Significant differences between treatments within the same species are indicated with *.

The results obtained demonstrated that both clove EO and pure eugenol at 300 μg/mL affected colony growth and the growth rates of all fungal species evaluated when compared to the control (only potato dextrose agar, PDA), with eugenol exhibiting the most potent antifungal activity (Table 2; Figure 1 and Figure 2).

*Rhizoctonia solani* was the fungus that grew most rapidly on PDA (y = 10.34 mm·day^−1^), followed by *Fusarium oxysporum* f. sp. *lycopersici* (y = 6.00 mm·day^−1^). In the presence of clove EO, the growth rate of *R. solani* was reduced (y = mm·day^−1^), and was completely inhibited with pure eugenol. *V. dahliae* was the slowest-growing fungus (y = 1.41 mm·day^−1^). Its growth rate was significantly reduced in the presence of clove EO (y = 0.60 mm·day^−1^) and completely inhibited with pure eugenol at 300 μg/mL. Additionally, the growth rates of *F. oxysporum* f. sp. *lycopersici* and *F. solani* f. sp. *cucurbitae* were reduced in the presence of clove EO (y = 1.62 mm·day^−1^ and 0.77 mm·day^−1^, respectively) compared with the growth rate of the control (y = 6 mm·day^−1^ and 3.99 mm·day^−1^, respectively). Further, both fungi were reduced with eugenol treatment, showing growth rates of y = 0.30 mm·day^−1^ and y = 0.40 mm·day^−1^, respectively (Table 2, Figure 1).

Interestingly, as shown in Figure 1, the presence of clove EO in the culture medium suppressed the early development of *V. dahliae*, with no visible growth observed until day 6. A similar inhibitory effect was noted for *F. solani* f. sp. *cucurbitae*, whose growth initiation was postponed until day 4 in the presence of clove EO, and without growth until day 6 with eugenol. Moreover, *F. oxysporum* f. sp. *lycopersici* delayed growth onset until day 2 with clove EO and until day 6 with eugenol. *R. solani* exhibited no visible growth until day 2 and no growth with eugenol.

According to our results, we emphasize the effect of eugenol in the medium where no growth occurred in *R. solani* or *V. dahliae* over the 7 days, and where the onset of growth in *F. oxysporum* f. sp. *lycopersici* and *F. solani* f. sp. *cucurrbitae* was delayed until day 6; after this, they exhibited very slow growth rates at y = 0.3 mm·day^−1^ and y = 0.4 mm·day^−1^, respectively.

Figure 2A illustrates the radial growth distribution under three conditions: control (PDA), clove oil (Cl), and pure eugenol (E) treatments. All fungal species, with the exception of *Alternaria alternata* (AA), exhibited significant growth inhibition when treated with eugenol (PDA vs. E). In the case of clove oil treatment, *A. alternata* (AA) and *Curvularia hawaiiensis* (CH) were the only species that did not show a statistically significant reduction in growth (PDA vs. Cl). A comparison between clove oil and eugenol treatments revealed no significant differences in most cases, indicating that both treatments exert an equally strong antifungal effect. Notably, for *F. oxysporum* f. sp. *lycopersici* (FOL), pure eugenol was exceptionally effective, resulting in complete inhibition of fungal growth. Statistical differences between treatments were computed using ANOVA, and Figure 2B presents the corresponding *p*-values obtained from the Tukey HSD test at a 95% confidence level, where significant differences are highlighted.

#### 2.2.2. Determination of Mycelial Growth Inhibition (MGI) of Clove EO and Pure Eugenol on Fungal Assay

**Figure 3:**
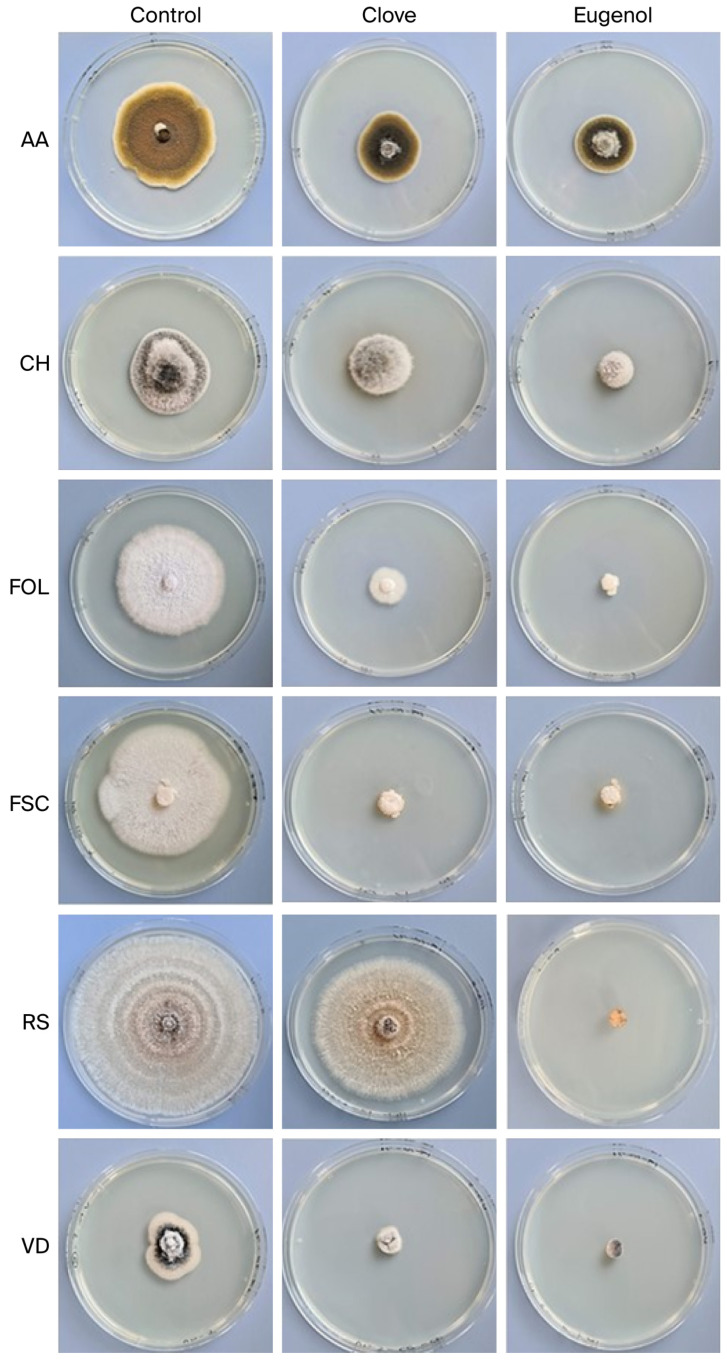
Effects of clove and eugenol essential oils (300 µg/mL) on colony diameter growth, and mycelial growth inhibition (MGI) of *Alternaria alternata* (AA), *Curvularia hawaiiensis* (CH), *Fusarium oxysporum* f. sp. *lycopersici* (FOL), *Fusarium solani* f. sp. *cucurbitae* (FSC), *Rhizoctonia solani* (RS), and *Verticillium dahliae* (VD) at 7 days.

Data obtained in the Section 2.2.1. corroborated the MGI parameter (Table 3, Figure 3). The highest MGI values with clove EO were obtained for *F. oxysporum* f. sp. *lycopersici* and *F. solani* f. sp. *Cucurbitae*, ranging from approximately 77 to 88%, respectively. Pure eugenol was particularly effective in reducing *F. oxysporum* f. sp. *lycopersici* and *F. solani* f. sp. *Cucurbitae,* with values of 94% and 95%, respectively, and completely inhibited the growth of *R. solani* and *V. dahliae* (100%).

*A. alternata* was the most resistant fungus to these treatments, followed by *C. hawaiiensis,* with MGI values of 38.59% and 34.35% at 7 days of inoculation, respectively, when treated with clove EO. However, the highest fungal inhibition values were obtained with eugenol (40 to 69%).

### 2.3. In Vivo Studies on the Melon C. melo cv. vedrantais and C. melo cv. makuwa for the Control of Fusarium oxysporum f. sp. lycopersici and Fusarium solani f. sp. cucurbitae

According to the results obtained, we have selected *F. oxysporum* f. sp. *lycopersici* and *F. solani* f. sp. *cucurbitae* for the in vivo tests because both species cause significant losses in melon, and clove EO and eugenol have demonstrated a significant antifungal effect against them. Additionally, both are producers of mycotoxins causing mycotoxin contamination in food.

**Figure 4:**
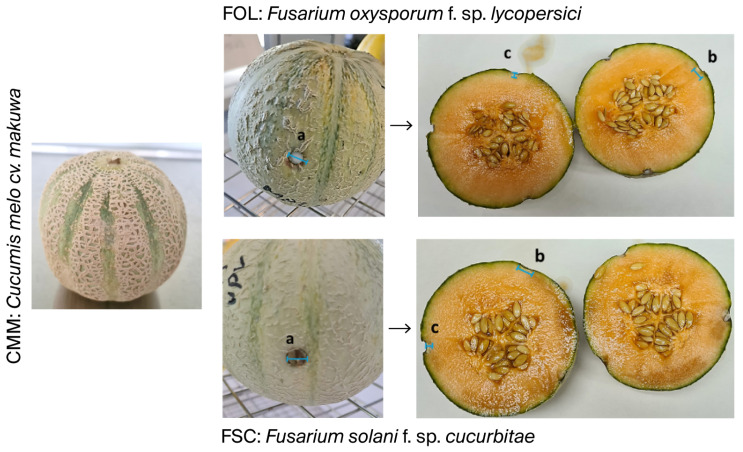
In vivo study of the resistance of the *Cucumis melo* cv. *makuwa* (CMM) to the growth inhibition of *Fusarium oxysporum* f. sp. *lycopersici* (FOL) and *Fusarium solani* f. sp. *cucurbitae* (FSC). Parameters measured: (a): external diameter of the fungal inoculation; (b): internal diameter of the damage caused by the inoculation; (c): internal depth of the damage caused by the inoculation.

As shown in Table 4, in vivo tests showed that in eugenol-rich melons (CCM), the fungal inoculation disc did not progress after 5 days of inoculation at 21 °C and 85% humidity (Figure 4). Notably, in these melons, we observed that the colony discs of *F. solani* f. sp. *cucurbitae* exhibited a reduction in size, likely due to dehydration, fungal mortality, or colony disc retraction. *F. solani* f. sp. *cucurbitae* did not developed necrotic spots or internal rot. Furthermore, *F. oxysporum* f. sp. *lycopersici* exhibited a minimal inhibition halo, scarcely perceptible to the naked eye, with external lesions measuring around 0.2 to 0.3 mm, and internal lesions displaying only a growth diameter of 0.3 mm and depths ranging from 0.2 to 0.5 mm (Figure 4).

**Figure 5:**
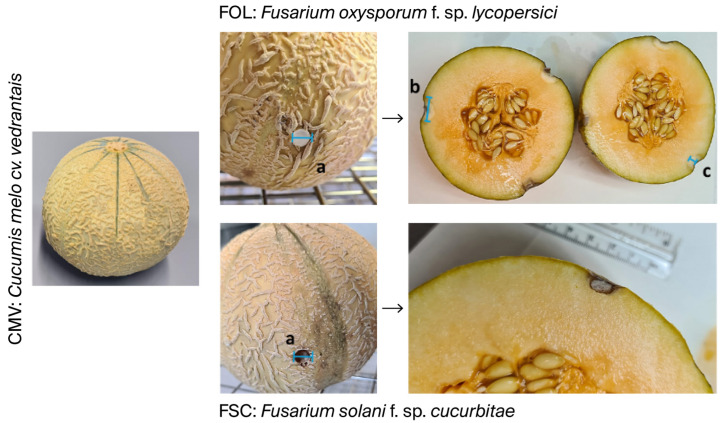
In vivo study of the resistance of the *Cucumis melo* cv. *vedrantais* (CMV) to the growth inhibition of *Fusarium oxysporum* f. sp. *lycopersici* (FOL) and *Fusarium solani* f. sp. *cucurbitae* (FSC). Parameters measured: (a): external diameter of the fungal inoculation; (b): internal diameter of the damage caused by the inoculation; (c): internal depth of the damage caused by the inoculation.

Meanwhile, the eugenol-poor *Cucumis melo* cv. *vedrantais* melons allowed the growth and development of both *F. oxysporum* f. sp. *lycopersici* and *F. solani* f. sp. *cucurbitae* fungi, producing external necrotic spots ranging from 13.8 to 15.5 mm in diameter and internal tissue rot measuring 14 to 15.2 mm in diameter and 8.7 to 12.8 mm in depth, with measures including the inoculation disc (Figure 5).

## 3. Discussion

Melon is widely consumed worldwide due to its sensory attributes and high nutritional value, serving as a rich source of essential vitamins and minerals with proven health benefits [7]. However, phytopathogens cause major losses in cultivation and postharvest stages. Postharvest decay is an important problem in melon production, largely caused by fungal pathogens, and currently lacks effective management strategies [11,12,13,14].

Different management strategies aim to prevent yield and quality losses in melon, with recent focus on eco-friendly alternatives. Agrochemical dips after harvest are common but pose health and environmental risks. Various additives and sanitizing agents have demonstrated antimicrobial properties [36]. Isothiocyanates have shown effective antimicrobial activity, reducing postharvest *Alternaria* rot by up to 88.67% [37]. Likewise, essential oils (EOs) have gained increasing attention due to their broad-spectrum biological activities. Essential oils are complex mixtures with only two to three major compounds at high concentrations. Clove EO, obtained from leaves, contains about 100 components, with eugenol being the predominant constituent, ranging from 30% to 95% [38]. One of the findings of the present study was the confirmation that the commercial clove leaf essential oil analyzed exhibited a high concentration of eugenol (89.28%), alongside smaller amounts of β-Caryophyllene (6.00%) and α-Humulene (1.68%), among other compounds consistent with previously reported data.

Several authors have demonstrated the potential of different EOs against fungal plant pathogens, including those obtained from species of the genera *Thymus*, *Mentha*, *Cinnamomum*, *Origanum*, *Laurus*, *Piper*, and *Citrus*, among others [21,22,23,24,25,29]. Extensive research has concentrated its studies on the genera *Alternaria*, *Botrytis*, *Fusarium*, *Penicillium*, and *Rhizoctonia*, making them the most frequently phytopathogenic fungal. Numerous studies have demonstrated that the antifungal response of a given phytopathogenic fungus can vary significantly depending on both the essential oil and the fungal species involved, highlighting the specificity of the interaction between the EO’s chemicals and the pathogen’s sensitivity [29]. In our work, we demonstrated that both clove EO and pure eugenol at 300 μg/mL reduced the fungal growth and the growth rates of six fungal pathogens of global significance in melon plants or fruits in the field or postharvest in in vitro conditions. Clove oil and eugenol exhibited notable antagonistic activity against *Alternaria alternata*, *Curvularia hawaiiensis*, *Fusarium oxysporum* f. sp. *lycopersici*, *Fusarium solani* f. sp. *cucurbitae*, *Rhizoctonia solani*, and *Verticillium dahliae*. Additionally, pure eugenol showed clear fungicidal effects against *R. solani* and *V. dahliae*, highlighting its potential as a natural agent for disease management. Consistent with our findings, previous works have demonstrated the antifungal effect of clove EO against *F. oxysporum* f. sp. *lycopersici* in in vitro assays [28,39]. Similar results were obtained in previous studies conducted by our research team, which demonstrated the antifungal activity of clove EO within a range of 100 to 300 μg/mL against four pathogenic fungi isolated from Mediterranean rice grains: *Alternaria alternata*, *Curvularia hawaiiensis*, *Fusarium proliferatum*, and *Fusarium oxysporum*. This activity was evident both in in vitro assays and when the oil was applied as a biofilm to rice grains, effectively reducing the development of all fungal pathogens [40]. The results showed that increasing the concentration led to higher levels of fungal inhibition, with a complete (100%) inhibition of *C. hawaiiensis* observed at 300 mg/L, while the other fungal species showed values of MGI between 54.80% and 64.94%.

The high eugenol content identified in the commercial clove EO used in this study (89.28%) may explain its strong antifungal activity, as previously reported by Santamarina et al. [31], who found a similar composition (88.58%) in clove oil effective at 300 mg/L against multiple fungal pathogens isolated from rice grains. These authors obtained values of MGI between 53% and 100% against *A. alternata*, *Botrytis oryzae*, *Fusarium graminearum*, *Fusarium equiseti*, and *Fusarium verticillioides*. In addition, Muñoz Castellanos et al. [41] evaluated the effect of clove EO with 85% eugenol against *F. oxysporum* and *Aspergillus niger* at different concentrations between 100 and 500 μg/mL. Their results showed that the antifungal effect was proportional to the increase in concentration, with total inhibition at 500 μg/mL. In this regard, eugenol has been widely recognized as the primary bioactive compound responsible for the antifungal properties of clove EO, and its concentration is closely related to the efficacy observed in vitro. These findings highlight the importance of characterizing the chemical composition of essential oils when evaluating their bioactivity, as variations in major constituents such as eugenol can significantly influence their effectiveness against phytopathogenic fungi.

Furthermore, recent studies have demonstrated the antifungal activity of clove EO and eugenol against *Candida* spp., which cause human candidiasis [38]. This is of particular relevance, since among the most common plant-contaminating fungi, such as *Bipolaris*, *Curvularia*, *Alternaria*, and *Fusarium*, pose high risk to human health after consumption [17,18,19]. In this sense, *Fusarium*-related rots have been reported globally and are considered a major constraint to the commercial distribution of melons [42]. According to Medeiros Araujo et al. [12], several *Fusarium* species have been isolated from commercial melon cultivars causing fruit rot, including members of the *Fusarium incarnatum–equiseti* species complex (FIESC)*,* the *Fusarium solani* species complex (FSSC)*,* the *Fusarium oxysporum* species complex (FOSC), and the *Fusarium fujikuroi* species complex (FFSC). *Fusarium* is one of the most important genera of phytopathogenic fungi worldwide and one of the most important mycotoxin-producing genera. *Fusarium*-produced mycotoxins can contaminate crops pre- and postharvest, posing health risks and acting as virulence factors that increase fungal pathogenicity. In this regard, *F. solani* f. sp. *cucurbitae* has been reported as a melon pathogen and deoxynivalenol (DON) producer [43], while *F. oxysporum* f. sp. *lycopersici* produces fusaric acid in infected tomato [44]. Thus, the dual role of the species of *Fusarium*, as both plant pathogens and mycotoxin producers, underscores the importance of reducing or avoiding *Fusarium* contamination in melon production systems for both yield protection and food safety.

Our results demonstrated the effectiveness of clove EO and pure eugenol against six phytopathogens. Therefore, the effect of the volatile compound eugenol against several phytopathogens is clear. Interestingly, in previous studies it has been demonstrated that some melons have high levels of eugenol among their volatile compounds, such as pocket melon (*Cucumis melo* ssp. *dudaim*), which are associated with the fruits’ aroma [32,33,35,45,46]. These melons with high levels of eugenol have attracted interest as a valuable genetic resource for melon breeding programmes. Disease resistance has been identified in these melons as viruses and *Fusarium* wilt, but these resistant traits are being incorporated into breeding programmes aimed at enhancing melon quality [35]. However, there is a lack of studies evaluating the role of endogenous eugenol in melon resistance to postharvest pathogens. In this regard, our study examined the resistance of melons *Cucumis melo* cv. *vedrantais* (CCV) and *Cucumis melo* cv. *makuwa* (CCM) chosen specifically for their contrasting eugenol levels against two pathogens, with CCM having a high concentration (~1940 mg/g), and CCV, in which eugenol was not detected. Also, we selected *F. oxysporum* f. sp. *lycopersici* and *F. solani* f. sp. *cucurbitae* among the six fungal pathogens evaluated in in vitro tests due to their importance as melon pathogens and mycotoxin producers. Our results showed that eugenol-rich melons inoculated with *F. solani* f. sp. *cucurbitae* did not develop necrotic spots or internal rot. In contrast, *F. oxysporum* f. sp. *lycopersici* caused only minimal external and internal lesions, with an average size of 0.2 mm. However, we registered a greater progression of rot symptoms in the CCV fruits. It is known that eugenol exhibits potent antifungal activity through a multimodal mechanism through which it disrupts the fungal cell membrane, as it is lipophilic and can penetrate the lipid bilayer of cell membranes, altering their permeability and causing ion leakage and loss of essential metabolites. Though inhibition of essential enzymes, eugenol can bind to membrane proteins and enzymes, denaturing key proteins for fungal metabolism, such as respiratory enzymes and cell synthesis. Additionally, it disrupts energy metabolism, since eugenol interferes with oxidative phosphorylation within fungal mitochondria, reducing ATP production. Finally, it produces oxidative stress, accelerating cell death [47,48].

To our knowledge, this is the first study to demonstrate that eugenol-rich melon fruits exhibit resistance to *F. oxysporum* f. sp. *lycopersici* and *F. solani* f. sp. *cucurbitae*, both of which are known for fruit decay. We believe that our research provides valuable insights for the management of diseases affecting melon, particularly fruit decay, either through fruit protection strategies or by breeding fruits with high eugenol content, encouraging the development of genetic improvement programmes focused on melon varieties rich in this bioactive compound.

Given the global need for food, the research conducted demonstrates the potential of clove essential oil and eugenol to reduce postharvest losses in melon fruit, while also reducing the use of agrochemicals, in the interest of more sustainable agriculture. The results obtained open a line of research for the development of cultivars that produce fruits with a high eugenol content and are resistant to *Fusarium* attack. It also opens the possibility of formulating biofilms that cover fruits and vegetables, protecting them from fungal losses.

## 4. Materials and Methods

### 4.1. Clove Samples and Eugenol

Guinama (La Pobla de Vallbona, Valencia, Spain) provided the commercial samples of essential oil extracted from clove leaves (*Syzygium aromaticum* L.), lot 9449600032, using natural mechanical methods, stating a eugenol content of 89%. Sigma-Aldrich (Madrid, Spain) supplied the pure eugenol. Both were stored at 4 °C until analysis and antifungal testing. These are both renowned and reliable companies.

### 4.2. Gas Chromatography–Mass Spectrometry (GC/MS)

GC–MS analysis was conducted using an Agilent 5973N system (Agilent Technologies Spain, SL, Barcelona, Spain) equipped with a capillary column coated with 95% dimethylpolysiloxane and 5% diphenyl (HP-5MS UI; 30 m length, 0.25 mm internal diameter, 0.25 μm film thickness). The oven temperature was programmed to hold at 60 °C for 5 min, then ramped at 3 °C/min to 180 °C, followed by an increase at 20 °C/min to 280 °C, where it was held for 10 min. Helium was employed as the carrier gas at a flow rate of 1 mL/min. Samples were introduced using the split injection mode with a 1:30 split ratio. Mass spectra were acquired over an *m*/*z* range of 30–500 at an ionization energy of 70 eV. Kovats retention indices (RIs) were calculated using co-injected standard hydrocarbons. Compound identification was based on mass spectral data and confirmed by comparison of their RIs, relative to C_8_–C_32_ n-alkanes, and mass spectra with authentic standards or reference data from the NIST 2005 Mass Spectral Library and relevant literature.

### 4.3. Fungal Species

The fungal species employed in this study were *Alternaria alternata* and *Curvularia hawaiiensis*, isolated in the Botany Laboratory of the Department of Agroforest Ecosystems (LBEA), Universitat Politècnica de València, Spain and deposited in the Colección Española de Cultivos Tipo (CECT) as CECT 20923 and CECT 20934, respectively; *Fusarium oxysporum* f. sp. *lycopersici* was provided by the CECT (CECT 2715); *Fusarium solani* f. sp. *cucurbitae* raza 1 (GIHF-145), *Rhizoctonia solani* (GIHF-192), and *Verticillium dahliae* (GIHF-327) were provided by the Collection of the Grupo de Investigación en Hongos Fitopatógenos, Instituto Agroforestal de Mediterráneo of the Universitat Politècnica de València (UPV), Spain. Fungi were preserved on PDA (potato dextrose agar) at 5 °C until further use.

### 4.4. Plant Material

The melons used in the work included *Cucumis melo* subsp. *melo* var. *cantalupensis* cv. *vedrantais* (CCV) and *Cucumis melo* subsp. *agrestis* var. *makuwa* cv. makuwa (CCM), provided by the Universitat Politècnica de València (UPV), Spain, and were harvested at commercial maturity. CCM had high eugenol contents (~1940 mg/g) and CCV had no detectable eugenol.

### 4.5. In Vitro Antifungal Effect of Clove Essential Oil (CEO) and Eugenol

The assay was carried out in Petri dishes (90 × 15 mm), dissolving 300 μg/mL of clove EO or eugenol supplemented with Tween 20 and 0.1% of clove essential oil in sterilized potato dextrose agar (PDA) at 45–50 °C while it was still in liquid form. Then, plates were inoculated with disks (8 mm diameter) of a 7-day-old colony of each tested fungus and incubated at 25 °C in the dark. Fungal growth was assessed daily by measuring colony diameters in two perpendicular directions for 7 days. To calculate the growth rates (mm·day^−1^), linear regression of the radius (mm) as opposed to the time (days) was carried out. Six replicates were prepared for each fungus. Control plates contained PDA added with 0.1% Tween 20 and the respective fungal isolate, without essential oil.

Mycelial growth inhibition (MGI) was determined on day 7 by the formula [49]

MGI = [(CD − OD)/CD] × 100

where CD is the average diameter of the colonies in the control plates and OD is the average of the colonies’ diameter in the plates with essential oil or eugenol.

### 4.6. In Vivo Studies on the Melon C. melo cv. vedrantais and C. melo cv. makuwa for the Control of Fusarium oxysporum f. sp. lycopersici and Fusarium solani f. sp. cucurbitae

The melons used for this study were grown at the Universitat Politècnica de València (UPV), Valencia, Spain. A total of 24 melons were evaluated: 6 melons each from two varieties and two fungal species. The melons were surface sterilized with a 3% sodium hypochlorite solution for 3 min, and then washed twice with sterile distilled water for 5 min and allowed to dry.

Next, using a sterile 6 mm corer, a 5 mm deep disc of melon skin was removed and replaced with a 6 mm diameter × 3 mm depth disc of each fungal species studied, grown for 5 days on PDA. Three inoculation points were performed per melon, with n (18) per treatment. The melons were incubated in a chamber with a humidity of 85% at 21 °C. On day 5, melon fruits were evaluated measuring the external diameter of the lesions (mm). Then, they were cut in half, and the diameter and depth of the internal lesion (mm) were measured.

### 4.7. Statistical Analysis

The effect of the clove EO against six fungal species was evaluated by an analysis of variance (ANOVA) for a completely randomized design using the Python v3.9, Scipy Stats module v1.15.1 (scipy.stats). Means were compared by the HSD Tukey test (*p* ≤ 0.05) available in the same Python package.

## 5. Conclusions

Our results demonstrate the potential of clove essential oil and pure eugenol as antifungal alternatives to conventional agrochemicals. Clove oil with 89.28% eugenol and pure eugenol reduced the growth of all fungal species tested, and both were particularly active against *Fusarium oxysporum* f. sp. *lycopersici*, *Fusarium solani* f. sp. *cucurbitae*, *Rhizoctonia solani*, and *Verticillium dahliae*, with a growth inhibition of 95 to 100% with pure eugenol.

Moreover, *Cucumis melo* cv. *makuwa* melon fruits with a high eugenol content were resistant to *Fusarium*-induced rot. In vivo tests showed that in eugenol-rich melons, the fungal inoculation disc does not progress after 5 days of inoculation at 21 °C and 85% humidity. Notably, in these melons, we observed that the colony discs of *F. solani* f. sp. *cucurbitae* exhibited a reduction in size, likely due to dehydration, fungal mortality, or colony disc retraction. This is particularly relevant for *Fusarium*, as current management strategies for diseases caused by this fungus and the rot it causes remain largely ineffective. These findings demonstrate the potential of clove essential oil and pure eugenol to reduce postharvest losses in melons and contribute to food security.

Furthermore, the results obtained open a line of research for improving melon production, with the future goal of developing cultivars that produce fruits with a high eugenol content, thereby extending their shelf life, and an improved aroma, given the high sensory value of this compound.

## Figures and Tables

**Table 1:** Chemical composition of clove essential oil.

Compound	RI *	Mean ± s. e. **
***Sesquiterpene hydrocarbons***		**8.06 ± 0.50**
β-Caryophyllene	1420	6.00 ± 0.39
α-Humulene	1455	1.68 ± 0.09
*allo*-Aromadendrene	1462	0.03 ± 0.00
γ-Muurolene	1477	0.02 ± 0.00
α-Muurolene	1497	0.03 ± 0.00
*trans*-Calamenene	1522	0.15 ± 0.01
δ-Cadinene	1521	0.11 ± 0.01
α-Calacorene	1543	0.04 ± 0.00
***Oxygenated sesquiterpenes***		**0.92 ± 0.05**
Caryophyllene oxide	1582	0.78 ± 0.04
Humulene epoxide II	1607	0.14 ± 0.01
***Aromatic compounds (C_6_–C_3_; C_6_–C_1_)***		**89.48 ± 0.33**
Eugenol	1357	89.28 ± 0.28
Dihydro Eugenol	1372	0.02 ± 0.02
Vanillin	1394	0.09 ± 0.02
Methyl Eugenol	1407	0.03 ± 0.00
4-Hydroxy-3-methoxy-Cinnamaldehyde	1728	0.07 ± 0.01
**Others**		**0.54 ± 0.15**
Monoacetin	1261	0.02 ± 0.01
Diacetin	1270	0.03 ± 0.01
Triacetin	1375	0.49 ± 0.13
**Total identified**		**99.54 ± 0.07**

* RI: retention index relative to C_8_–C_32_ n-alkane on HP-5MS column; ** Values are means ± standard deviation of three samples.

**Table 2:** Effect of clove essential oil and eugenol on radial growth (mm) and growth rates (mm·day^−1^) of *Alternaria alternata*, *Curvularia hawaiiensis*, *Fusarium oxysporum* f. sp. *lycopersici*, *Fusarium solani* f. sp. *cucurbitae*, *Rhizoctonia solani*, and *Verticillium dahliae*.

Species–Treatment	Mean *	GR
*A. alternata*-PDA	18.53 ± 11.36	5.62 (0.99)
*A. alternata*-Cl	11.14 ± 7.05	3.47 (0.98)
*A. alternata*-E	9.15 ± 5.91	2.91 (0.98)
*C. hawaiiensis*-PDA	9.59 ± 3.62	1.74 (0.95)
*C. hawaiiensis*-Cl	5.91 ± 2.46	1.18 (0.94)
*C. hawaiiensis*-E	3.14 ± 0.94	0.45 (0.92)
*F. oxysporum* f. sp. *lycopersici*-PDA	20.50 ± 12.11	6.00 (0.99)
*F. oxysporum* f. sp. *lycopersici*-Cl	4.52 ± 3.23	1.62 (0.95)
*F. oxysporum* f. sp. *lycopersici*-E	0.53 ± 0.89	0.30 (0.08)
*F. solani* f. sp. *cucurbitae*-PDA	13.92 ± 8.08	3.99 (0.99)
*F. solani* f. sp. *cucurbitae*-Cl	1.21 ± 1.29	0.77 (0.78)
*F. solani* f. sp. *cucurbitae*-E	0.39 ± 0.65	0.40 (0.23)
*R. solani*-PDA	40.01 ± 20.85	10.34(0.99)
*R. solani*-Cl	13.74 ± 11.01	2.44 (0.98)
*R. solani*-E	0.00 ± 0.00	0.00 (0.00)
*V. dahliae*-PDA	4.51 ± 2.89	1.41 (0.97)
*V. dahliae*-Cl	0.56 ± 0.93	0.60 (0.36)
*V. dahliae*-E	0.00 ± 0.00	0.00 (0.00)

* Mean: mean radius ± standard error; GR: growth rate (R^2^); Cl: clove essential oil; E: pure eugenol; PDA: potato dextrose agar. Confidence interval with a probability of 0.95.

**Table 3:** Mean growth (mm) and mycelial growth inhibition (MGI) percentage for each fungus grown on PDA (control), PDA–clove (Cl) essential oil, and PDA–eugenol (E) at 7 days after incubation.

Treatment	AA	CH	FOL	FSC	RS	VD
**Control**	71.00	29.40	76.90	51.10	142.40	17.90
**Cl**	43.60	19.30	18.20	6.20	61.70	4.50
**E**	35.70	9.20	4.00	3.10	0.00	0.00
**MGI (%)**						
**Cl**	38.59	34.35	76.33	87.87	56.67	74.86
**E**	49.72	68.71	94.80	93.93	100.00	100.00

AA: *Alternaria alternata*; CH: *Curvularia hawaiiensis*; FOL: *Fusarium oxysporum* f. sp. *lycopersici*; FSC: *Fusarium solani* f. sp. *cucurbitae*; RS: *Rhizoctonia solani*; VD: *Verticillium dahliae*; Cl: Clove; E: Eugenol.

**Table 4:** Means and standard deviation (mm) of the *Fusarium oxysporum* f. sp. *lycopersici* (FOL) and *Fusarium solani* f. sp. *cucurbitae* (FSC) inoculation points in melons of the *Cucumis melo* cv. *vedrantais* (CMV) and *Cucumis melo* cv. *makuwa* (CMM) varieties on the fourth day of incubation at 21 °C and 85% relative humidity.

SAMPLE	FUNGUS	Point a (mm)			Point b (mm)			Point c (mm)		
		Exterior	Interior		Exterior	Interior		Exterior	Interior	
		Diameter	Diameter	Depth	Diameter	Diameter	Depth	Diameter	Diameter	Depth
**CMV**	**FOL**	15.5 ± 1.5	15.2 ± 1.7	8.7 ± 2.0	14.5 ± 0.8	14.3 ± 1.8	9.5 ± 3.3	13.8 ± 1.1	14.2 ± 0.8	11.8 ± 1.0
	**FSC**	14.4 ± 1.2	14.2 ± 1.7	11.7 ± 1.6	14.7 ± 1.3	14.0 ± 1.9	12.8 ± 1.7	14.2 ± 0.9	14.5 ± 1.0	10.7 ± 1.2
**CMM**	**FOL**	6.3 ± 0.5	3.0 ± 0.0	3.2 ± 0.4	6.2 ± 0.4	3.3 ± 0.5	3.5 ± 0.5	6.3 ± 0.5	3.3 ± 0.5	3.3 ± 0.5
	**FSC**	6.0 ± 0.0	3.0 ± 0.0	3.0 ± 0.0	6.0 ± 0.0	3.0 ± 0.0	3.0 ± 0.0	6.0 ± 0.0	3.0 ± 0.0	3.0 ± 0.0

Discs of inoculation: 6 mm diameter × 3 mm depth.

## Data Availability

The raw data supporting the conclusions of this article will be made available by the authors on request.

## Abbreviations

The following abbreviations are used in this manuscript:

AA	*Alternaria alternata*
CECT	Colección Española de Cultivos Tipo
CCM	*Cucumis melo* subsp. *agrestis* var. *makuwa* cultivar *makuwa*
CCV	*Cucumis melo* subsp. *melo* var. *cantalupensis* cultivar *vedrantais*
Cl	Clove essential oil
CH	*Curvularia hawaiiensis*
DON	Deoxynivalenol
GC/MS	Gas Chromatography–Mass Spectrometry
E	Pure eugenol
EO	Essential oil
FOL	*Fusarium oxysporum* f. sp. *lycopersici*
FSC	*Fusarium solani* f. sp. *cucurbitae*
FIESC	*Fusarium incarnatum–equiseti* species complex
FSSC	*Fusarium solani* species complex
GIHF	Grupo de Investigación en Hongos Fitopatógenos, Instituto Agroforestal de Mediterráneo of the Universitat Politècnica de València
MGI	Mycelial growth inhibition
NIST	National Institute of Standards and Technology
PDA	Potato dextrose agar
RIs	Retention indices
RS	*Rhizoctonia solani*
VD	*Verticillium dahliae*
